# Postextinction Geographies: Audiovisual Afterlives of the Bucardo and the Ivory-Billed Woodpecker

**DOI:** 10.1080/24694452.2024.2304206

**Published:** 2024-03-11

**Authors:** Hannah Hunter, Adam Searle

**Affiliations:** a Department of Geography and Planning, Queen’s University , Canada; b School of Geography, University of Nottingham , UK

**Keywords:** 动物遗迹, 收藏, 灭绝, 超人类地理, 技术中介, colección, extinción, geografías más-que-humanas, mediación tecnológica, postvidas animales, animal afterlives, collection, extinction, more-than-human geographies, technological mediation

## Abstract

How do technologies animate more-than-human geographies after extinction? How can geographical scholarship evoke, or bring presence to, extinct biota? In an epoch simultaneously characterized by biotic loss at an unthinkable scale and the increased presence of representations depicting nonhuman life through mass media and digitization, we examine the epistemic, affective, and ethical possibilities of extinct animal traces to shape more-than-human geographies. We show how technological apparatuses inaugurate afterlives of extinction troubling binaries of extinct–extant and absence–presence. Specifically, we consider audio and visual remains of two taxa producing awkward and unsettling postextinction geographies: the ivory-billed woodpecker and the bucardo. Sound recordings and other historical traces continue to forge contemporary connections between human searchers and the ivory-billed woodpecker, although no sighting of the ghost bird has been universally accepted since 1944. The bucardo was declared extinct in 2000, but it was tentatively reanimated through a failed 2003 cloning project; in this milieu, visual technologies and representations conjure alternative presence and speculative futures beyond technoscientific spectacle. Through conversing our own situated, speculative, and technologically mediated relations with these taxa—and situating the technological assemblages themselves—we present some of the lively, contested, and dispersed ways technological apparatuses affect and inaugurate animated geographies after extinction.

## Abstract (zh)

技术如何激活灭绝后的超人类地理？地理研究如何唤起或展现已灭绝的生物群落？目前, 生物灭绝的规模大得难以想象。与此同时, 越来越多的大众媒体和数字化描绘了非人类生命。我们研究了灭绝动物痕迹在认知、情感和伦理方面的可能性, 并塑造了超人类地理。展示了技术装置如何开启灭绝动物的遗迹, 挑战了灭绝与存活、不存在和存在的二元认知。具体的, 我们考虑了两个类群(象牙喙啄木鸟和布卡多)的音频和视觉遗迹, 质疑了灭绝后地理。尽管自1944年以来就没有发现这种魔鬼鸟的踪迹, 录音和其它历史遗迹继续建立了人类搜索者和象牙喙啄木鸟的联系。布卡多在2000年被宣布灭绝, 但它们在2003年一个失败的克隆项目中暂时复活。在这种背景下, 视觉技术和表现方式唤起了技术科学奇迹之外的另类存在和推测性未来。通过探讨我们特定的、推测性的、技术中介的与这些类群的关系, 并考虑各种技术, 我们呈现了技术装置影响和激活灭绝地理的一些有效的、争议性的和分散式的途径。

## Abstract (es)

¿Cómo animan las tecnologías a las geografías más-que-humanas después de la extinción? ¿Cómo puede la erudición geográfica evocar o darle existencia a la biota extinta? En una época que simultáneamente se caracteriza por la pérdida biótica a una escala impensable, y la creciente presencia de representaciones que muestran la vida no humana a través de los medios masivos y la digitalización, examinamos las posibilidades epistémicas, afectivas y éticas de las huellas de animales extintos para configurar las geografías más-que-humanas. Mostramos cómo los aparatos tecnológicos inauguran las posvidas de la extinción cuestionando los binarios de lo extinto–existente y de la ausencia–presencia. Específicamente, tomamos en consideración los restos sonoros y visuales de dos taxones que producen geografías postextincionales hoscas e inquietantes: el pájaro carpintero pico de marfil y el bucardo. Las grabaciones de sonidos y otros relictos históricos siguen forjando conexiones contemporáneas entre las búsquedas humanas y el pájaro carpintero pico de marfil, así desde 1944 no haya sido universalmente aceptado avistamiento alguno de esta ave fantasmal. El bucardo se declaró extinto en 2000, pero fue tentativamente revivido por medio de un fallido proyecto de clonación en 2003; en este entorno ambiental las tecnologías y representaciones visuales conjuran una presencia alternativa y unos futuros especulativos más allá del espectáculo tecnocientífico. A través de nuestras propias relaciones situadas, especulativas y tecnológicamente mediadas con aquellos taxones –y situando los propios ensamblajes tecnológicos– presentamos algunas de las formas vivas, controvertidas y dispersas en que los aparatos tecnológicos afectan e inauguran geografías animadas después de la extinción.

The static crackle of the audio file palpably resonates, providing an ambient sonic backdrop. It is punctuated, intermittently, by the toy-trumpet-like yelps of a ghost bird now largely thought to be extinct.

A fleeting glance at the lens from behind a rock. Within seconds the ibex leaps out of frame, once again a ghost story, yet it leaves traces of this moment, immortalized through photographic technology.

Through the circulation of technological representations and affective encounters, extinct nonhumans persist as palpable afterlives. How can geographers and cognate social scientists research, relate to, and represent these absent creatures made knowable through audiovisual technologies? Inspired by extinction studies, the environmental humanities, and more-than-human geography, we examine how technological mediation shapes extinction’s afterlives and unsettles biotic absence. We take inspiration from philosopher Vinciane Despret’s pivotal writing on death, assemblage, and attunement to nonhuman worlds to contemplate how, where, and to what effects “the dead interfere in the lives of the living” (Despret 2021d, 8). Through tracing the collection and circulation of extinction’s audiovisual afterlives, we story some of the ways that technological apparatuses animate two seemingly absent species, inaugurating lively postextinction geographies.

Extinction is an ending of collective worlds and ways of life, but it also a place of beginnings: Abundant work across the environmental humanities considers the worlds produced in the wake of contemporary extinctions (e.g., van Dooren 2014, 2019; Despret 2017; Rose, van Dooren, and Chrulew 2017; Garlick 2019a; Wrigley 2020, 2023; Guasco 2021). Boundaries between absence and presence, life and death, and beginnings and ends are not always clear, however. This article is preoccupied with two animals posing awkward questions around extinction’s ontological formation due to complex more-than-human relations mediated by contemporary and historical technological assemblages: the bucardo (*Capra pyrenaica pyrenaica*) and the ivory-billed woodpecker (*Campephilus principalis*). We explore the technological assemblages surrounding these species’ extinctions as performative apparatuses of reanimation, troubling binaries, and forming relations in extinction’s wake.

The bucardo and the ivory-billed woodpecker are animals on different edges of extinction and fruitful cases in conversation. The bucardo, a subspecies of Iberian ibex endemic to the Pyrenees, was officially declared extinct in 2000 but has since been subject to numerous de-extinction projects, including a brief—ultimately unsuccessful—revival in 2003, when the extinct taxon was cloned in Spain (Folch et al. 2009). The lone U.S. representative of the *Campephilus* woodpecker genus, the ivory-billed woodpecker,^1^ was last “officially” sighted in 1944, but has become a canonical “ghost species” ever since—subject to a cascade of reported sightings, sound recordings, and blurry video captures of varying persuasiveness (T. Gallagher 2005; McCorristine and Adams 2020).

Both cases speak to practices of collection and representation reflective of the technological, historical, and cultural milieu in which extinction narratives are situated. Both are archetypical of “nonhuman charisma,” receiving disproportionate sociocultural attention and conservation funding before and after their generally accepted “extinctions”—a condition we understand here as postextinction charisma (Lorimer 2007). Both continue to affect contemporary humans seeking to understand their absence and pose ethical questions concerning their care. Both were the empirical subject of our respective doctoral projects, yet known to us solely through technological mediation. Such relations are interpersonal and contextually bound, yet we build on these connections and those of others to consider how technological assemblages conjure specific afterlives with continued political potentials.

“Extinct things do not just disappear,” wrote Jørgensen (2022a, 217). Human relations with extinct animals have always flourished through encounters with mediated traces, such as oral histories and fossil records, which animate and are animated by those who encounter them, be they conservationists, artists, activists, or geographers. The recently extinct species discussed here, however, are also survived by distinct kinds of affective and mobile traces. Contemporary audiovisual technologies of collection (productive of material-semiotic traces evidencing nonhuman existence) and circulation (mediating and diffusing relations with such traces) have exploded and multiplied their afterlives. Such technologies produce novel ecologies, more-than-human relations, and knowledges through their ability to sense, mediate, and speculate with traces of extinction scattered across time and space. For the ivory-billed woodpecker and bucardo, whose perceived absence or presence is profoundly unsettled, these diffuse and fractured traces have had profound consequences.

Despret’s philosophy provides a fruitful conceptual framing to examine postextinction geographies. Her work has illustrated how various epistemic communities use technologies to forge relations with the dead and how varied techniques bring presence to absence (Despret 2015, 2019). Mediations between the living and dead occur through “distance reduction technologies,” which work temporally and spatially; whether the labor of a spirit medium or paleontologist—for example—humans deploy a range of tools to bring presence to absence and investigate the dead. “They are convocation apparatuses [*dispositifs de convocation*]” (Despret, 2019, 7). Most theoretical readings of apparatus [‘*dispositif*’] prominent in Foucault’s scholarship have “emphasized the orderly over the generative” (Legg 2011, 131). Despret’s *dispositif*, though, is closely aligned with Deleuze’s (1992, 159) philosophy where an apparatus is “a tangible, multilinear ensemble” that continually forges novel relations through the creative production of subjectivities. In following audiovisual apparatuses and the contemporary postextinction geographies they animate, we engage the broader assemblages that produce material and semiotic traces, including, but not limited to, historical and contemporary more-than-human relations, knowledge practices, and affects.

Our focus on technological apparatuses in the coauthoring of extinction’s afterlives recognizes that extinction is necessarily always technologically mediated (Jørgensen 2022b). Technologies—and their capacity to produce representations, experiences, and affects—mediate these relations. They are politically charged, as technologies produce situated and partial perspectives (Haraway 1988). In speculating on postextinction geographies, as authors we both “become with” and “start from” the animals we study. To paraphrase Despret’s (2013a) writing on assemblage and historical animal encounters, our articulation “*enacts* as well as *being enacted by* new narratives, new assemblages, which in turn activate each of the beings involved, and involve more beings in a cascade of practices” (37). For Despret (2013a), the assemblage is “an active process of attunement that is never fixed once and for all” (38).^2^ Technologies affect and—to some extent, capture—animals while still alive, shaping the capacity of historical animals to affect and be affected. Convocation apparatuses, then, are the product and component part of an assemblage whose collective power animates extinction’s afterlives, and individual human, nonhuman, and technological agencies can only be understood within this assemblage.

To engage convocation apparatuses is to “re-member: both ‘recompose’ and ‘recall’ which keep a memory alive” (Despret 2021b). Despret (2019) argued that both the desire to re-member the dead and the dead’s desire to be re-membered hold together in an assemblage; “ontological priority” cannot be given to the living or dead. To re-member “demands that we be capable of responding to, and answering for, that which we inherit” (Despret 2016, 178). Relations with extinct animals therefore inaugurate novel forms of responsibility: In ethical terms, they shape how humans respond to, represent, and remember extinction, and create instances where power, knowledge, and accountability can be questioned. Responsibility thus concerns *“making the* (living) *body available*” for affective relation (Despret 2013b) through technological mediation.^3^ Responsible relations with extinct species, then, are “not just about being in mourning, but *actively keeping existences present*” (Despret 2021b).

Maintaining the dead in the world, for Despret (2021d), is of paramount geographical concern, for “the dead has to be situated; that is, a place has to be ‘made’ for them” (10). Situating extinct species involves creating situations in which encounters can emerge, obliging geographers to forge connections through sparse temporal and spatial configurations. In this article, we both narrate and formulate these postextinction geographies through employing writing practices that explore assemblages of postextinction reanimation. These writings are empirical and ethnographic, but also speculative, as they not only articulate but compose spaces of ongoing presence and relation (Despret 2013a, 2021a). As such, speculation is not about “breaking with ‘reality’ but seeking to make it perceivable, making aspects of this reality thinkable and feel-able” (Stengers 2006, in Despret 2021d, 79). Speculative geographies, and the technologies implicated and instrumentalized in their practice, do not therefore provide a clean passage to the past but bridges to alternative worlds. Following Salazar (2017, 166), we mobilize speculation as “an ethnographically inflected,” empirically grounded method.

“To create stories, to make history, is to reconstruct,” for Despret (2016, 178), “in a way that opens other possibilities for the past in the present and the future.” We thus consider relations with extinct nonhumans as speculative gestures toward worlds-to-come, rather than as historical artifacts. In doing so, we follow a promise of geography as a means of “imagining, inhabiting, and producing alternative worlds” (Dekeyser 2023). We specifically examine visual and sonic technologies as means of perceiving—and producing—worlds, grounded through the empirical cases of our respective studies.

Our argument elaborates as follows. First, we outline methodological approaches that inform our practice of storying postextinction geographies through technological mediation. We then turn to sonic technologies and mediated relations with the ivory-billed woodpecker and its position as an animal of disputed extinction, before elaborating the visual circulations of the bucardo and its candidacy for techno-fix de-extinction projects. To conclude, we consider these cases together and examine the postextinction relations enabled by and experienced through technological mediation. Ultimately, we demonstrate the animating and political potential of audiovisual afterlives, which inaugurate lively charismatic subjects and geographies with distinct consequences to “extinction” as an event and an experience. Along the way, we illustrate the potentials of geographical research to bring presence to extinct animals both as a tool of investigation and representation, in part by bringing Despret’s work further into conversation with geographical scholarship on death and nonhuman extinction.

## Methodology: Composing Presence in Geographical Research

Death and extinction are not interchangeable objects of study: The former feels more personal and intimate, the latter profound and collective. To understand postextinction geographies shaped by absence and mediation, though, we can learn from extant geographical work on death. Considerable social scientific work has problematized life as a discrete object of study (e.g., Povinelli 2016; TallBear 2017), focusing on meaningful, affectively resonant, and ethically charged relations occurring with bodies that cease to be living. Burgeoning studies of death, dying, and the afterlife in human geography have centered the concept of “deathscapes”: the “spaces and landscapes of emotional intensity through which the dead continue to be ‘with’ us” (Romanillos 2015, 561; see also Maddrell and Sidaway 2010; Heng 2022). Following these interventions, we geographically situate the practices through which the dead and the living connect in the context of animal extinction, in parallel to historical geographical approaches attentive to relations with archival traces (Cameron 2001). Thus, stories of the dead “do not enchant the world, but resist its deanimation. They do not fight against absence but compose with presence” (Despret 2021d, 126). They hold potential to engage multispecies worlds past, present, and future (Searle 2020a).

The question of how to story past animal lives responsibly is central to environmental history and extinction studies (Fudge 2002; Benson 2011; Cortés Zulueta 2015; Jørgensen 2017). To what extent can historical animals be “known,” given that they do not often leave the kinds of documents that traditional historical inquiry is accustomed to? For Derrida (2006), nonhumans and humans both produce material-semiotic traces in the world, thus thinking about historical animals means considering “a multiplicity of organizations between living and dead” (31). Extinction’s traces, then, continue to shape how more-than-human worlds are experienced and known.

Animal traces are found in traditional archives—bugs squashed between pages, animals in correspondence—or outside—animals have left traces written in landscapes, architecture, cultures, and ecologies (see, e.g., Lorimer 2006; Benson 2011; Oliver 2021; Searle 2021; Bersaglio and Margulies 2022). Taking animal traces and their persistent relevance seriously is our methodological approach to tell deliberately endless stories of and with the dead (see Lorimer 2019; Despret 2021d). We engage animal traces as a means of experimentation to elucidate extinction’s afterlives. Indeed, following Debaise (2020), these stories are already present in material-semiotic traces, “but it is up to us to articulate them, to intensify their meaning and to accompany the possibilities they carry” (17).

Extinct animal traces, however, are not usually abundant. Patchett and Foster’s (2008, 106) investigation of the blue antelope thus advocates for “make-do methods” of “repair work,” wherein “diffuse historical fragments” of past animals can be assembled in unconventional and illuminating ways (see also Patchett, Foster, and Lorimer 2011). Similarly, through considering Scottish ospreys’ place-making practices, Garlick (2019a) suggested “speculative ethology” for constructing “a more lively account of past ecologies” (228). Such approaches take what they can from historical animal traces—in archives, landscapes, literature, oral history, or otherwise—and enliven these with the help of contemporary ethological knowledge, insights from critical animal studies, and engagements of creative and artistic fabulation. The goal here is not necessarily to present historical facts of animals’ lives, but to tell stories in “speculative, risky and creative ways” (Garlick 2019a, 228) that recognize past animals’ agency and subjectivity (Pearson 2013; van Dooren and Rose 2016; Garlick 2019b; Howell and Kean 2019).

Geographic work on deathscapes also enriches our understanding of postextinction geographies in their exploration of how death affects the emotional and material production of space. For instance, Pitas and Shcheglovitova (2019) argued that both human and nonhuman deaths are affected by and affective of uneven urban geographies, and Maddrell (2016) demonstrated that bereavement can be a spatial process, where each person’s experience of grief can be individually mapped on physical, embodied-psychological, and virtual registers. Such studies emphasize the integral but multivarious role of space and place before, during, and after death, and how these relate to wider geographical processes. Relatedly, Garlick and Symons (2020, 299) recently outlined an agenda for geographies of extinction, advocating for “an explicit political ecology of extinctions-in-place.” Like Despret, they argued that foregrounding place helps to account for the site-specificities of extinction events, and how these relate to wider political and economic forces such as colonialism and capitalism. Most relevant to our study, they consider “geographies after extinction” which—aligned with geographic work about more-than-human spectrality and haunting (e.g., McCorristine and Adams 2020; Searle 2022)—accounts for “how particular places, sites or landscapes bear traces of absence in the wake of ecological destruction” (Garlick and Symons 2020, 313). Bersaglio and Margulies’s (2022) notion of “extinctionscapes” similarly considers how the absence-presence of extinction shapes landscapes long after the expiration of a species—in their case study of rhinos in Kenya, this takes the form of postextinction encounter value.

Geographical scholarship can further examine the dispersed and affective technological afterlives of extinct animals. Following the spirit of geographical traditions of “following the thing” (Cook 2004) and McCormack’s (2010) cultural geographic appropriation of “remote sensing,” we argue that storying the collection, circulation, and effects of particular technological traces offers unique insights into not only their entanglement with wider structures and processes, but also an expanded perspective of the ways in which extinct animals continue to make a difference in the world.

Heng’s approach to deathscapes is provocative in this vein. Through a visual ethnography of death and spirits in Singaporean Chinese religion, Heng (2022) argued that objects can be “material proxies of consociation,” wherein the dead “actively impact the decisions and actions of the living” (417). This raises an important question for extinction’s traces: Are the animate afterlives we explore evidence of extinct animals exerting spectral agencies through material proxies (Heng 2022), or are the traces (the “objects”) agential beings in and of themselves (e.g., Ash, Gordon, and Mills 2023)? Indeed, to follow and story these traces—as captures of animals before their death—is to prompt further questions about the biopolitics of representation (Yusoff 2012) and whether the absent animal is merely (and selectively) represented in such traces or reconstituted in them. We understand these technological afterlives as assemblages: dynamic (and at times problematic) coproductions of various humans, animals, and technologies, that nonetheless evidence ongoing agency of extinct animals in that they continue to affect the lives of the living within wider circulations of a “distributed field of affective materials” (McCormack 2010, 650).

In focusing on how specific audiovisual technologies are complicit in the coproduction of material-semiotic afterlives of extinction, we must ask how mediated postextinction geographies affect how more-than-human worlds are considered, crafted, and controlled. Indeed, if “the world dies from each absence; the world bursts from absence” (Despret 2017, 219), it is toward the latter we turn our investigation.

## Speculative Presents: Ivory-Billed Woodpecker

### Ethnographic Observations by Hannah Hunter, Collaboratively Written

It is July 2021, and today is my first interview with an “ivorybill hunter”: individuals searching the Southeastern United States for proof of the ivory-billed woodpecker’s presence. I thank Matt Courtman, former lawyer and cofounder of the grassroots group Mission Ivorybill, for making the time, and mutter an apology about not being an ivorybill expert. “None of us really are,” he assures me. Knowledge about ivorybills is limited and fractured and involves an awkward stitching together of traces: paintings by colonial naturalists; decaying taxidermy mounts; written observations in twentieth-century field books; and a crackly series of photos, videos, and sound recordings from 1935. The only comprehensive study of ivorybills by James Taylor Tanner in the 1930s occurred when the remnant population was already dwindling, and no sighting of the bird in the United States has been universally accepted since 1944 (Tanner 1942; Fitzpatrick et al. 2005). Although imaginations of the bird’s appearance seem close to reality—calibrated by at least 400 museum specimens—its behavior, range, and cause of decline remain fiercely debated (T.Gallagher 2005; Hill 2007; Snyder 2007; Gandy 2022).

For most, attempts to “know” the bird involve crafting stories through these fragmented material-semiotic deathscapes. Entering into these relations forces one to re-member (Despret 2016)—acknowledging the difficulty of completely “knowing” historical animals (Fudge 2002), and the necessity of creativity in these encounters (Patchett and Foster 2008). For me, the need for creative composition was compounded during COVID-19–related restrictions, where encounters were further mediated through online digitization: photos of taxidermy mounts, clipped and spliced audio recordings, and scanned field journals. Each of these pieces reflects practices of “technonatural history” shaped by the cultural and historical context they emerge from and are encountered within (Searle et al. 2023). Ivorybill traces are thus intrinsically entangled with technological histories of natural history, something itself shaped by the White, colonial gaze (Das and Lowe 2018). Representations of ivorybills are thus neither whole nor neutral. The species as understood through these heterodox narratives, then, is a situated, speculative construction.

Of course, there are many relatively unknown rare and extinct species due to the recent advent of technological capacities to record, observe, and story biology. Most life on Earth falls into this category and can only be known through speculative engagements with unknown pasts, often informed by scientific practices such as paleontology (Bastian 2020).^4^ For the ivorybill, though, epistemic unknowns have distinct consequences: The species is not yet “officially” extinct, and passionate ivorybill searchers still stalk the Southeastern bottomland forests, hoping to capture tangible evidence of the species’ survival. Despite proclaimed ivorybill rediscoveries (e.g., Fitzpatrick et al. 2005), “irrefutable evidence” like a clear photograph remains lacking. Instead, searchers offer an assemblage of fragments calibrated with fractured ivorybill traces: “credible” sightings matching naturalists’ field descriptions; photos of nesting cavities matching Tanner’s 1942 report; blurry videos of a bird’s tailing edges that match taxidermic mounts; and sound recordings matching other *Campephilus* species in South America, or ivorybill sounds captured in Louisiana in 1935 (T. Gallagher 2005; Hill 2007; Collins 2011; Latta et al. 2023). The same fractured assemblage of ivorybill traces informs search methods on the ground: instructing searchers, often in conflicting and ambiguous ways, where and how ivorybills might be found (Hunter 2023a).

Following a controversial 2021 recommendation of the U.S. Fish and Wildlife Service to officially declare the species extinct, Mission Ivorybill has held regular online meetings to discuss the speculative presence of ivorybills, trading evidence, testimonies, and advice for ivorybill searching. Two things are clear in these meetings. First, the disparate and contradictory fractures that shape multiple conceptions of ivorybills leave copious room for debate concerning speculative ivorybill presents, concerning where a hypothetical extant ivorybill would be found and what it would do, look like, and eat. Second, the mobile nature of ivorybills’ technological afterlives has both fueled and democratized the search for the species. More on this later.

**Figure 1.**
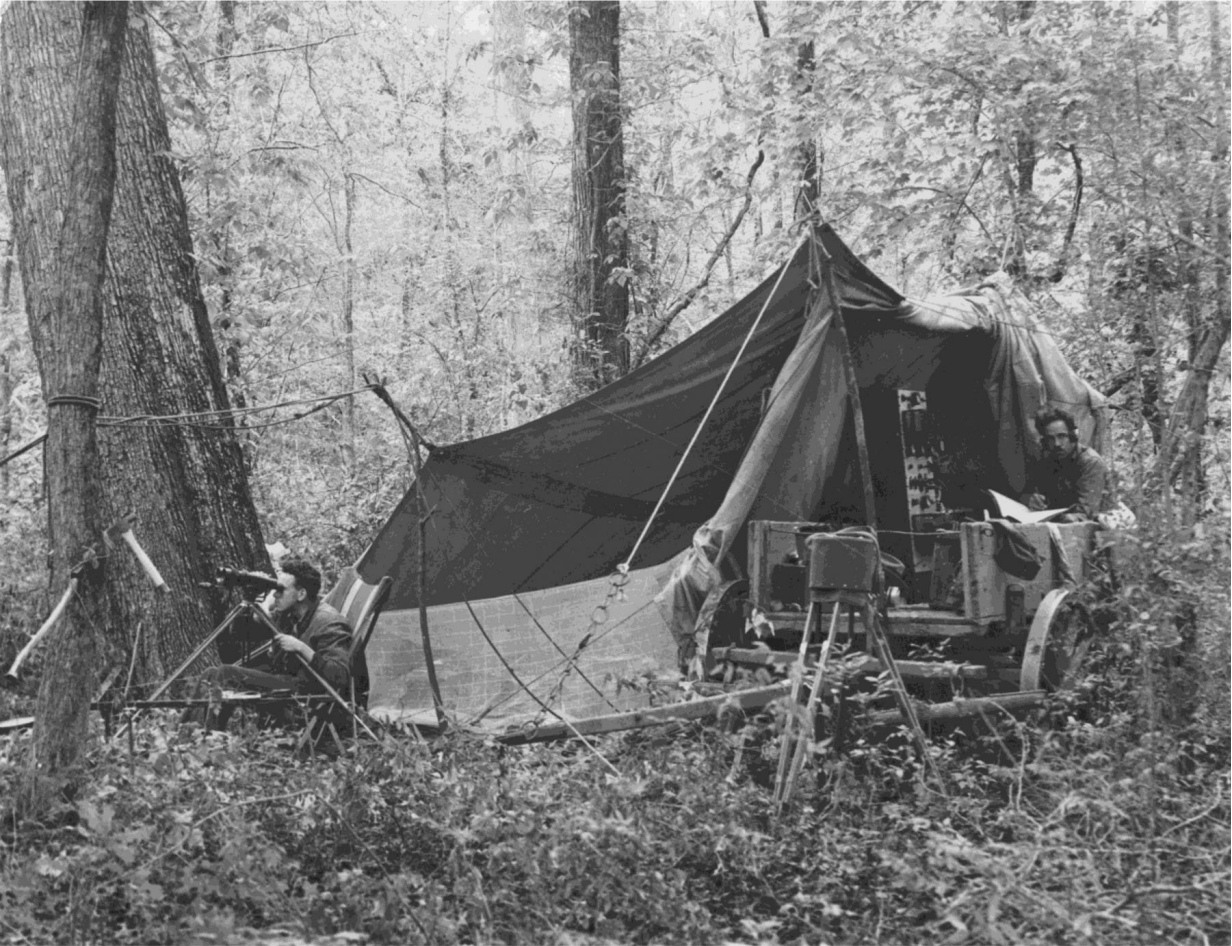
The ivory-billed woodpecker observation and recording camp, set up in the Singer Tract of Louisiana in April 1935. Albert Rich Brand papers, #21-18-1255. Division of Rare and Manuscript Collections, Cornell University Library.

There are two ivorybill sound recordings available on the Cornell Lab of Ornithology’s Macaulay Library Web site—the most recent was captured in Texas in 1968, and is considered controversial in its authenticity (Hardy 1975). The other series was captured in 1935 by a small team of Cornell ornithologists in the swamp forests of Louisiana (Figure 1)—it is these that I, and many others, listen to again and again.^5^ “LNS Catalogue Number 6784” an archivist declares at the start.^6^ The ivorybill cuts in, abruptly, with a disarming nasal joust called a “kent” call, a sound frequently described to resemble a toy trumpet or the sound of blowing through a clarinet mouthpiece (not a clarinet—a clarinet mouthpiece; this matters for your sonic imagination). The ten-minute recording oscillates between an individual bird and a mating pair heard in heated discussion, piercing through the crackles of the movietone sound camera reels. The juxtaposition of audibly outdated technology and the odd vocalizations of these birds produces an otherworldly affect, even without the knowledge of their definite death and presumed extinction.

**Figure 2.**
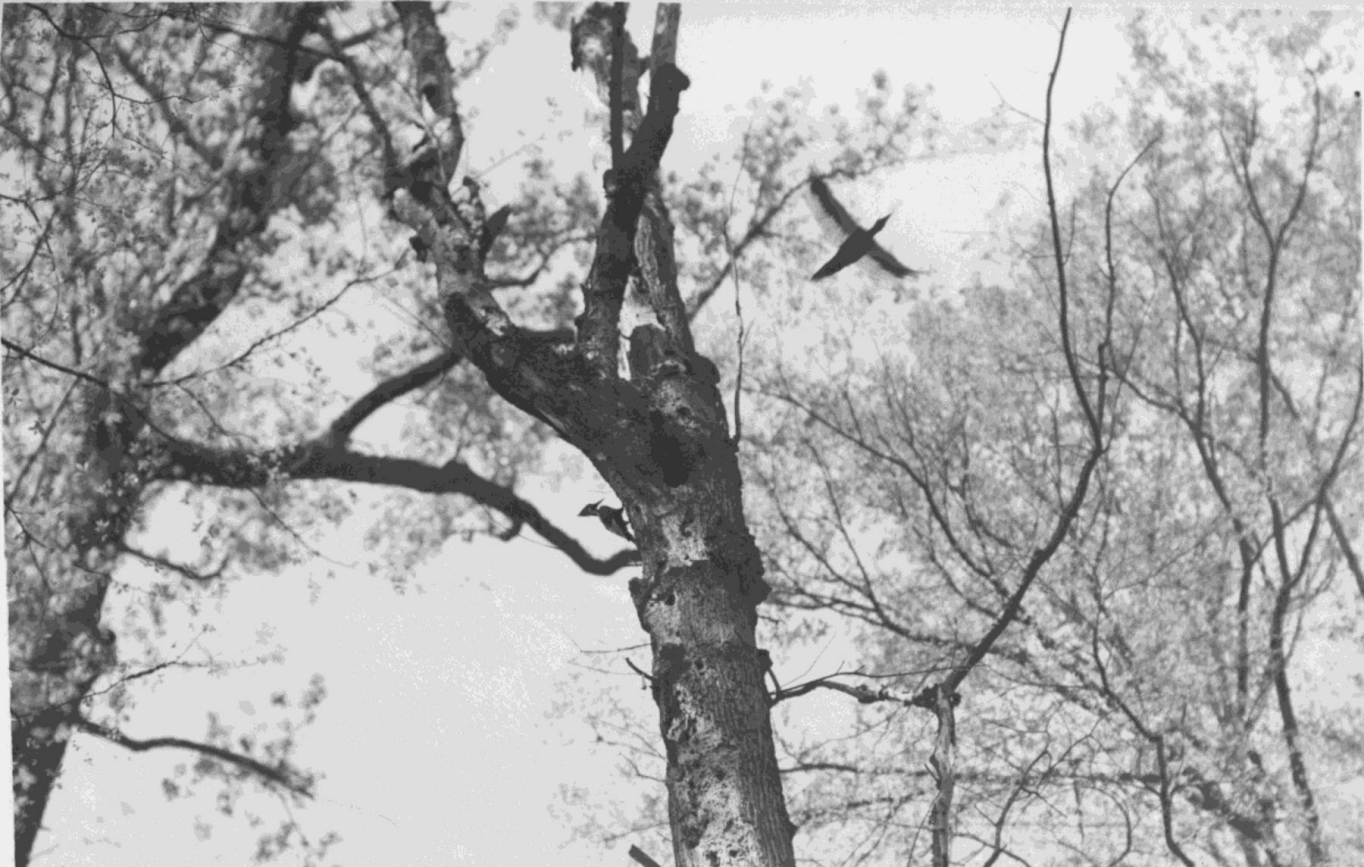
One of the only accepted photographs of ivory-billed woodpeckers, captured in Louisiana in 1935. These are the same individuals heard in the 1935 sound recordings. Albert Rich Brand papers, #21-18-899. Division of Rare and Manuscript Collections, Cornell University Library.

Sometimes the recordists are heard in the background. “I have to raise my hand to keep them from going in,” says someone faintly, revealing that this is not necessarily a “natural” ivorybill performance, but a choreographed assemblage of scientific intentions, technologies, humans, and nonhumans. In the accompanying expedition field notes, scientists explained they would scare the ivorybills out of nests to observe and record them (Allen and Kellogg 1937). Indeed, many now argue that the sounds on the 1935 tape are atypical, chronicling nesting birds irritated by the continuous presence of the recordists (T. Gallagher 2005). At 3:14, the ivorybill pair engage in “social chatter.” We hear the birds call to each other, drum at a tree, and one fly away. The field notes explain this behavior happens when the pair change guardianship of the nest while the other searches for food (Allen and Kellogg 1937). These sounds, when re-membered with the expedition notes, photographs (Figure 2), and videos, immerse listeners in this moment: a pair of lost birds fighting for survival. When the recordists returned to the nest cavity later in the month, the birds had disappeared.

There are scant sound recordings of presumed extinct bird species, thus ivorybill material is significant in shaping how lost biota are related to. What does it mean to listen to—to have access to—the sounds of birds that might never be heard again alive? The 1935 expedition that captured the ivorybills’ voice was one of the first of its kind, organized soon after portable wildlife sound recording became possible. This expedition explicitly sought to record the sounds of America’s “vanishing birds” before it was, presumably, too late—a sonic gift to future scientists, publics, and their imaginations (Brand 1935; Hui 2022). When listening to these ivorybills, the disjunction of absence and presence is palpable. The sound-makers here are now absent; so, too, perhaps, are all other makers of this sound. The recordists are dead, the technologies obsolete, the forest felled, and the original physical recording lost. Listening to ivorybills is a sonic iteration of Rose’s (2012) concept of double death, wherein mass extinction extinguishes not just bodies but intergenerational knots, uncoupling “the partnership between life and death” (128).

Yet in this deathscape and soundscape of cascading absence, there is presence. The recordings act as “distance reduction technologies” (Despret 2019) between contemporary listeners and historical birds. Scholars have drawn attention to the intensely transformative power of sound as a relational, embodied event transforming relations between bodies (M. Gallagher 2016; Born 2019). Sound recordings can, however, disorient, as recording technologies do not capture “sounds”, “but arrangements of charged particles in the semiconductive materials of solid state ‘flash’ memory, or the magnetic surfaces of hard drives, tapes, and minidiscs” (M. Gallagher 2015, 569). Upon playback, there is a “paradoxical spatiality” (M. Gallagher 2015, 574), as listeners vibrate with both “acoustic traces” (M. Gallagher 2015, 574) of the past, and “an ensemble of machines, here in the present” (M. Gallagher 2015, 574). In this way, playing historical sound recordings is a bringing-to-life or mediated speculation of ivorybill voices in spatiotemporalities otherwise absent of ivorybill voices. Recordings thus do not just sonify something lost, but sound something into presence. On pressing “play,” I do not conjure “actual” ivorybills, but co-create an ever-evolving assemblage of humans, machines, birds, places, and stories. Within this mediated and fractured field, I am listening closely, entwined, if only briefly, and if only partially, with a species otherwise so very far away.

Different people, in different places, at different times, have differently affected and been affected by these sound recordings, leading to distinct speculative rearticulations of ivorybills. The captures have been elevated in the context of the species’ contested extinction, most strikingly in the practice of playback, wherein ivorybill hunters play out the 1935 recordings in potential habitats in the hope that surviving ivorybills will respond. These open-access digitized recordings have been broadcast in ivorybill habitats by field scientists (Hill 2007) and grassroots ghost hunters (Courtman personal communication 2022). Many have also used spectrograms (visual representations) of the 1935 recordings in their searches in attempts to identify remnant populations through their sounds (e.g., Fitzpatrick et al. 2005; Latta et al. 2023). Some claim to have encountered extant ivorybills by carefully using historical traces of presence through methods like these (Hunter 2023a). Searchers hope these efforts could lead to conclusive evidence of the birds’ presence that, in turn, could ultimately save ivorybills from extinction.

**Figure 3.**
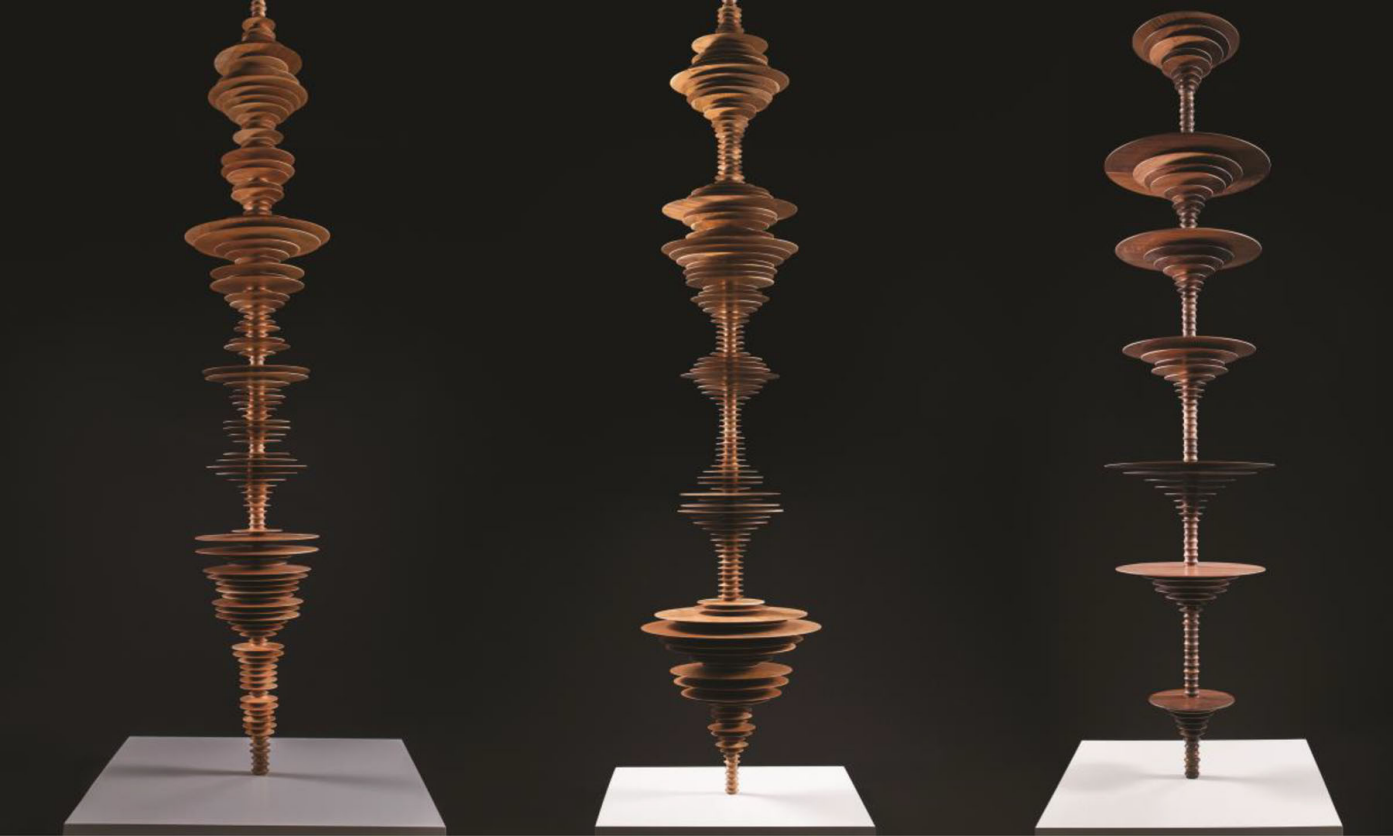
A selection of wooden sculptures from artist Elizabeth Turk’s (2020) series *Tipping Point: Echoes of Extinction*, Hirschl & Adler Modern Gallery. Each sound column represents the calls of an extinct, endangered, or recovering species—here (from left to right), brown pelican, bald eagle, and ivory-billed woodpecker. Image courtesy of the artist.

The sounds have also inspired artists grappling with extinction, where they are heard and rearticulated as tragic reminders of our broken ecology. For instance, American artist Elizabeth Turk’s (2020) *Tipping Point: Echoes of Extinctio*n series includes a wooden sculptural representation of the 1935 recording’s acoustic waveform structure (Figure 3), evocatively taking up space no longer occupied by the ivorybill calls themselves (Hunter 2023b). The affective afterlives of these recordings are multivarious: cocreating speculative presences of ivory-billed woodpeckers and working, in diffuse but entangled ways, to extend and profoundly alter the event of this species’ extinction (see McCorristine and Adams 2020).

Like many traces of past animals, however, these sonic fragments were not collected harmlessly. Not only are field recording practices linked to wider structures of colonial extractivism (Traisnel 2020; Hui 2022; Kanngieser 2023), but some have speculated that it was the proximity and interventions of recordists that drove those remnant ivorybills away from their nest in 1935 (M. Michaels, personal communication, 2022). Indeed, the colonial culture of collection, in part, led to the ivorybills’ decline in the first place: Although the birds’ bills and feathers had long been used by Indigenous groups, hunting in the nineteenth and early twentieth century by predominantly White settler scientists and commercial collectors, combined with habitat loss, reduced the population significantly (Tanner 1942; Snyder 2007; Gandy 2022). As Gandy (2022) argued, the disappearance of the species in eastern Arkansas was part of the systematic assault against nature bound up with the White masculinity of settler colonialism and the plantation system. An ahistorical, placeless conjuring of ivorybills through technology thus potentially occludes these histories, including the fact that the very trace facilitating ongoing relation itself was likely collected in a way that disturbed some of the last individuals of the species. If convocation apparatuses create worlds, what worlds are destroyed under such conditions?

Indeed, Gandy (2022) argued that a “white nationalist myth” of these landscapes as “‘unpeopled’ wilderness” (376) motivates contemporary ivorybill conservation discourses. In this view, the continued speculative presence of ivorybills is not necessarily positive, but this discourse does not define all postextinction ivorybill geographies. For instance, a letter by the Cherokee Nation in response to the U.S. Fish and Wildlife Service extinction recommendation notes the important role that this species holds in Cherokee culture and asks “the Service to continue conservation and investigative efforts to assure the protection of any individuals remaining” (Hoskin 2022). In sum, technological convocation is not an ahistorical, acultural, or normative endeavor. Traces are collected, encountered, and reanimated in place. Presence remains, relations flourish, and worlds continue to be made.

## Speculative Futures: Bucardo

### Ethnographic Observations by Adam Searle, Collaboratively Written

**Figure 4.**
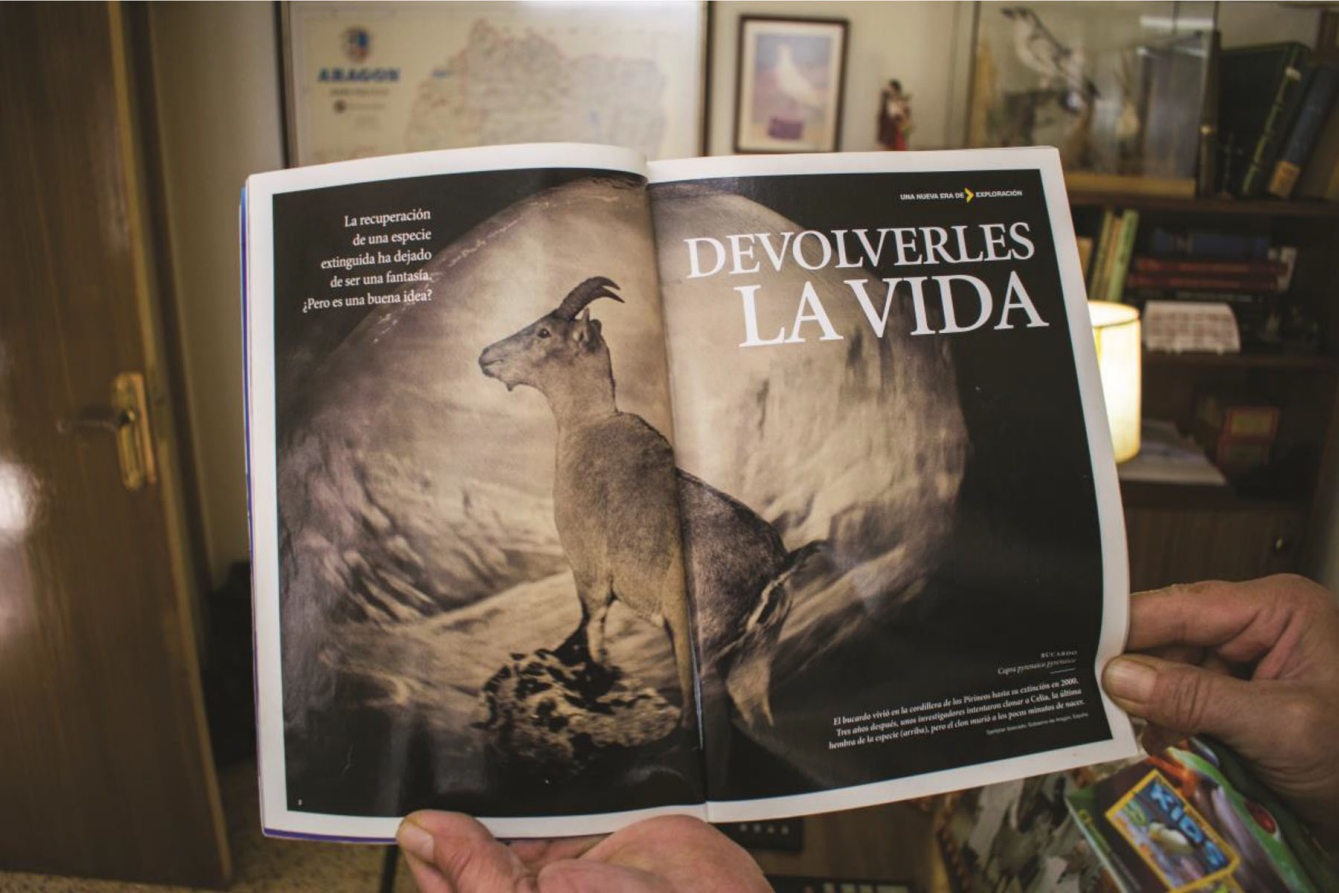
*National Geographic* magazine featuring the choreographed image of bucardo taxidermy, as shown to me by the taxidermist who stuffed the last bucardo in his studio.

The first bucardo that affected me was a photograph in *National Geographic* (Figure 4). It shows a speculative scene: A taxidermic specimen stands in profile, gazing over an artificial backdrop. Through drawing together various dead, inanimate objects, the image conjures a certain liveliness. It is made possible by multiple technologies, some now obsolete, including but not limited to the biotelemetry collar fitted to the animal while alive, without which her body would have been disappeared, eaten by scavengers and detritivores and lost to legend; the preservative chemicals and artificial skin mount that constitute the taxidermy; the camera; the printing apparatus sharing this vision in magazines around the world; and the Internet, further disseminating and maintaining this image in circulation.^7^ The complex technological assemblage articulating this image spans diverse spatiotemporal scales to transgress life and death.

“Bringing them back to life,” reads the article’s title, detailing contemporary aspirations to “resurrect” extinct biota through the application of biotechnologies like cloning or genome editing (Searle 2020b). These technoscientific imaginaries often foreground culturally prominent extinct fauna—mammoths, passenger pigeons, and thylacines. The bucardo, alongside, is a lesser known protagonist. After briefly recounting the bucardo’s 2000 extinction, the article adds, “On July 30, 2003, a team of Spanish and French scientists reversed time. They brought an animal back from extinction, if only to watch it become extinct again” (Zimmer 2013, 28). I was fascinated. Zimmer conceded that when scientists had briefly reanimated the bucardo, their techniques were “in hindsight, woefully crude”; yet should more advanced technologies be applied to other animals, extinction’s future permanence is in doubt. Those supporting this aspiration commonly called de-extinction often illustratively deploy the bucardo as evidence that deextinction is a technical plausibility (e.g., Church and Regis 2012) or that this ethical boundary has been crossed (see Kasperbauer 2017). I wanted to hear the stories of those who had lived alongside the animal both in its Pyrenean habitat and subsequently in laboratories, to collect stories of bucardo extinction and reanimation and, importantly, gain proximity to the bucardo myself.

Bucardo historically ranged the entire Pyrenean massif, from the Basque Atlantic to the Catalan Mediterranean, across the contemporary nation states of Andorra, France, and Spain. Yet centuries of overhunting and habitat exploitation drove bucardo to near extinction by the turn of the twentieth century. When the last bucardo was alive in 1999, a team of ecologists in the Ordesa Valley captured her to preserve her cells (Folch et al. 2009; Fernández Arias 2017; Crampe 2020). Like the 1935 Louisiana expedition, these scientists were attempting to capture an aspect of bucardo vitality to speculate on potential futures—here, facilitated through biotechnologies that were yet to be invented. This genetic material was cryopreserved in three different locations, and when the last bucardo died on 6 January 2000, it became the first extinct taxon outlasted by its cryopreserved cells (Searle 2020b). Novel modes of biopolitics were facilitated by this event in which “liveness can persist as posthumous after the event of death” through technological mediation, further blurring the distinctions between dead and alive (Colombino and Giaccaria 2016, 1046). Three years later, the bucardo clone was born and died in a laboratory on the industrial outskirts of Zaragoza, facilitated through technoscientific practice. These scientists were dreaming of, one day, repopulating the Pyrenees with the extinct bucardo (Folch et al. 2009; Fernández-Arias 2013). Subsequent cloning efforts failed, and the bucardo remains extinct. Yet it remains in the Pyrenees, woven into the cultural landscape (Searle 2022).

Years later, I went to Spain in search of the bucardo’s ghosts. These ghosts became the protagonist of my doctoral research, and my life reoriented around Pyrenean spectral ecologies. During ethnographic work in the village of Torla, which overlooks the Ordesa Valley near the Spain–France border where bucardo used to wander freely, I came across countless images of the last bucardo individuals. The images are speculative evocations of absent biota. For Roberts (2013), “images haunt between the visible and invisible, real and virtual, as material objects and abstract cognitive, embodied, subjective processes” (387). A photographed bucardo is neither wholly absent nor present; through the medium of photography, absences are made present, conjuring haunted landscapes and ecologies while rendering extinction affectively palpable (Searle 2022; Adams, McCorristine, and Searle 2023). These representations of the animal continue to forge new meanings and understandings of extinction and are entities that affect those who encounter them across spatial and temporal scales; to borrow Despret’s term again, they are “distance reduction technologies.” Although it emerges from a distinct spatiotemporal point, whenever a photograph is encountered it has the capacity to affect in particular ways each time different from the last. Through time, then, images of bucardo carry different meanings as they actively shape worlds in altered social and technical contexts. Since these encounters were captured, de-extinction has gained significant public and scientific attention, and the bucardo’s image is circulated in distinct discursive frames to either support or critique the scientific, ethical, and moral truth claims to de-extinction as a legitimate practice.

**Figure 5.**
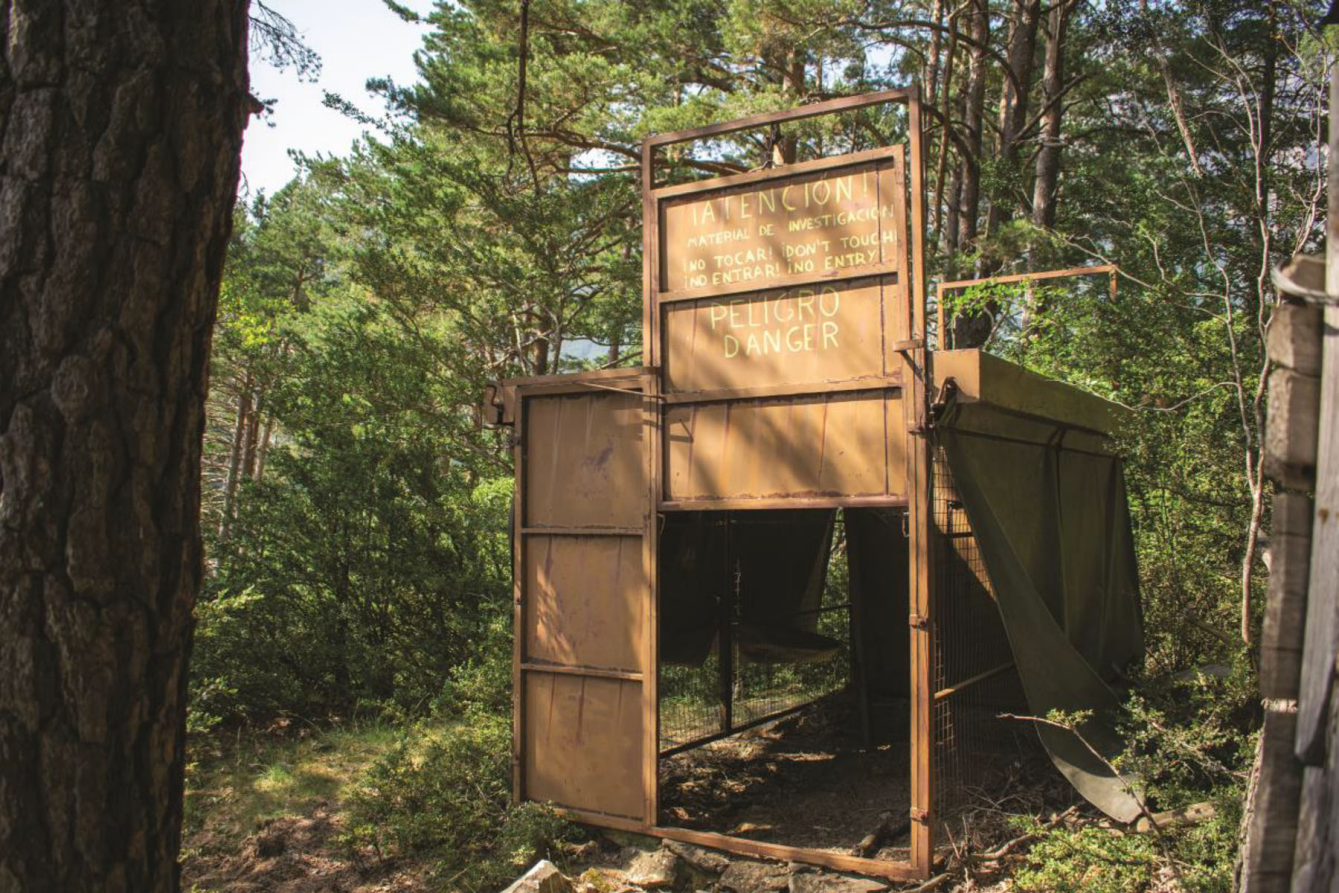
A disused bucardo trap in Ordesa, which is in a state of ruin. I finally encountered this trap after years of searching for it in July 2022.

I forged a profound relationship with the bucardo without ever encountering a living animal. I have encountered photographic and videographic imagery, and I have conducted years of ethnographic research in the bucardo’s footsteps to reanimate the landscape with my own embodied creations of these lost beings. As much as the bucardo’s extinction haunts the Pyrenees, so, too, does my speculation, facilitated and distorted by the circulation of imagery and mediation. In spending days on end in the Ordesa Valley searching for a ruined, obsolete bucardo trap (Figure 5), each rustle in the thick vegetation would trick my imagination into constructing a landscape populated by the ghost animal, aided by the images I had observed for years prior. Mediated relations reanimate the Pyrenean landscape, just as some scientists hope biotechnologies one day will.

The most famous photographs of the bucardo were taken by Bernard Clos (1985) in the Valle de Ordesa. They profoundly contrast the image in *National Geographic*—rather than a statuesque taxidermic specimen purveying the artificial valley, these individuals are seen entangled with their environment; the scenes they portray resemble fleeting encounters. Clos’s photography followed me throughout the Pyrenees. Each time these images re-membered a different history and allowed for people to narrate their own bucardo extinction stories and animate absences.

**Figure 6.**
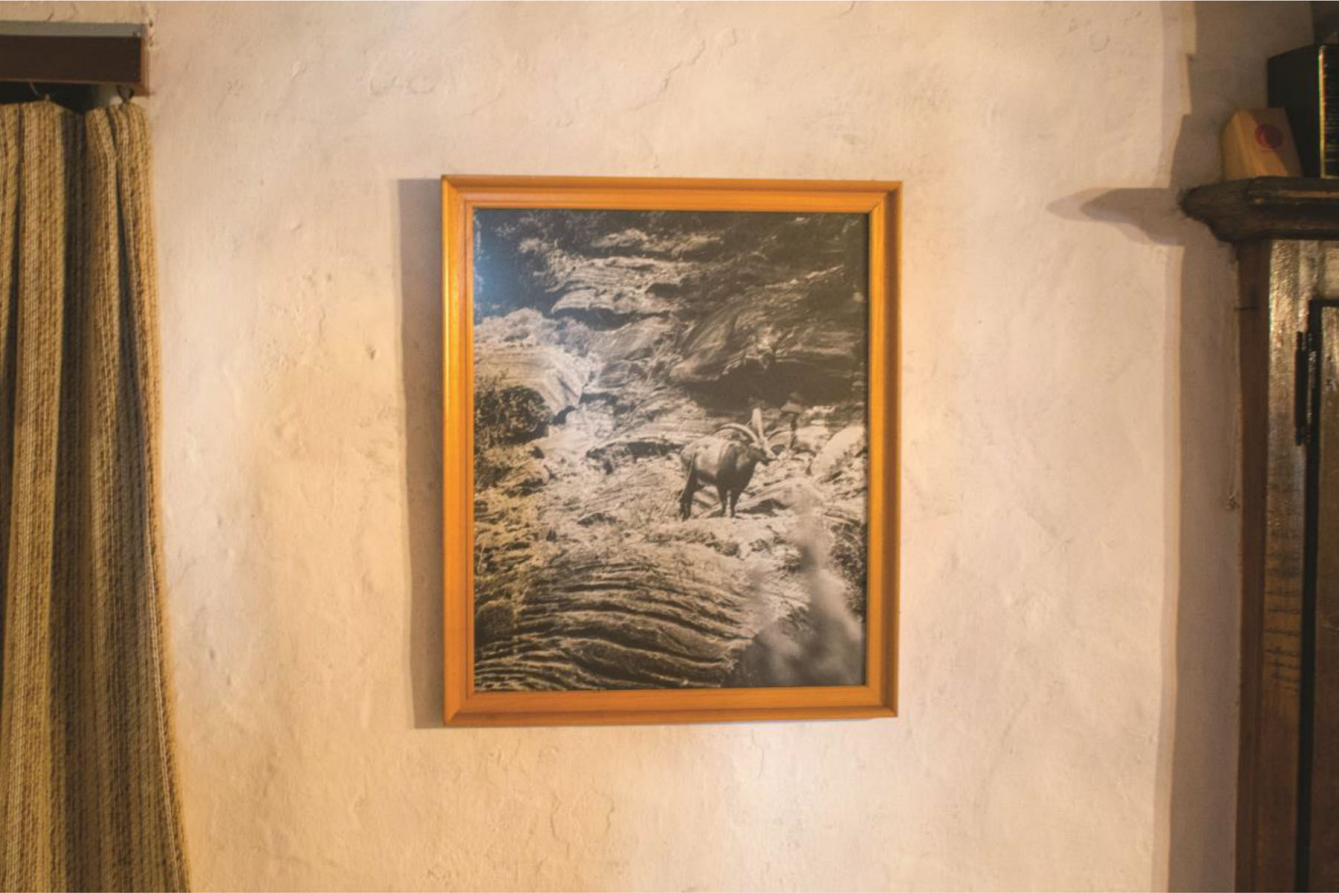
Photographed bucardo by Bernard Clos, hanging in Juan’s bedroom in Torla.

On the walls of a Medieval house in Torla hangs one of Clos’s photographs depicting bucardo from afar. Juan, who is showing me around, was a park ranger in the Ordesa National Park during the decades preceding the bucardo’s extinction. Juan spoke of this photo as a “memory” or “memento/souvenir” [*recuerdo*]. Juan added that it reminds him “how special seeing a bucardo was. In 1982, the same year as this photo, there were twelve bucardo. Every day there were less. This photo reminds me of when they used to leave the valley, to eat grass and sunbathe” (Figure 6). The photograph both tells a story and allows Juan to tell stories, animating aspects of the bucardo’s behavior in situ with personal detail.

**Figure 7.**
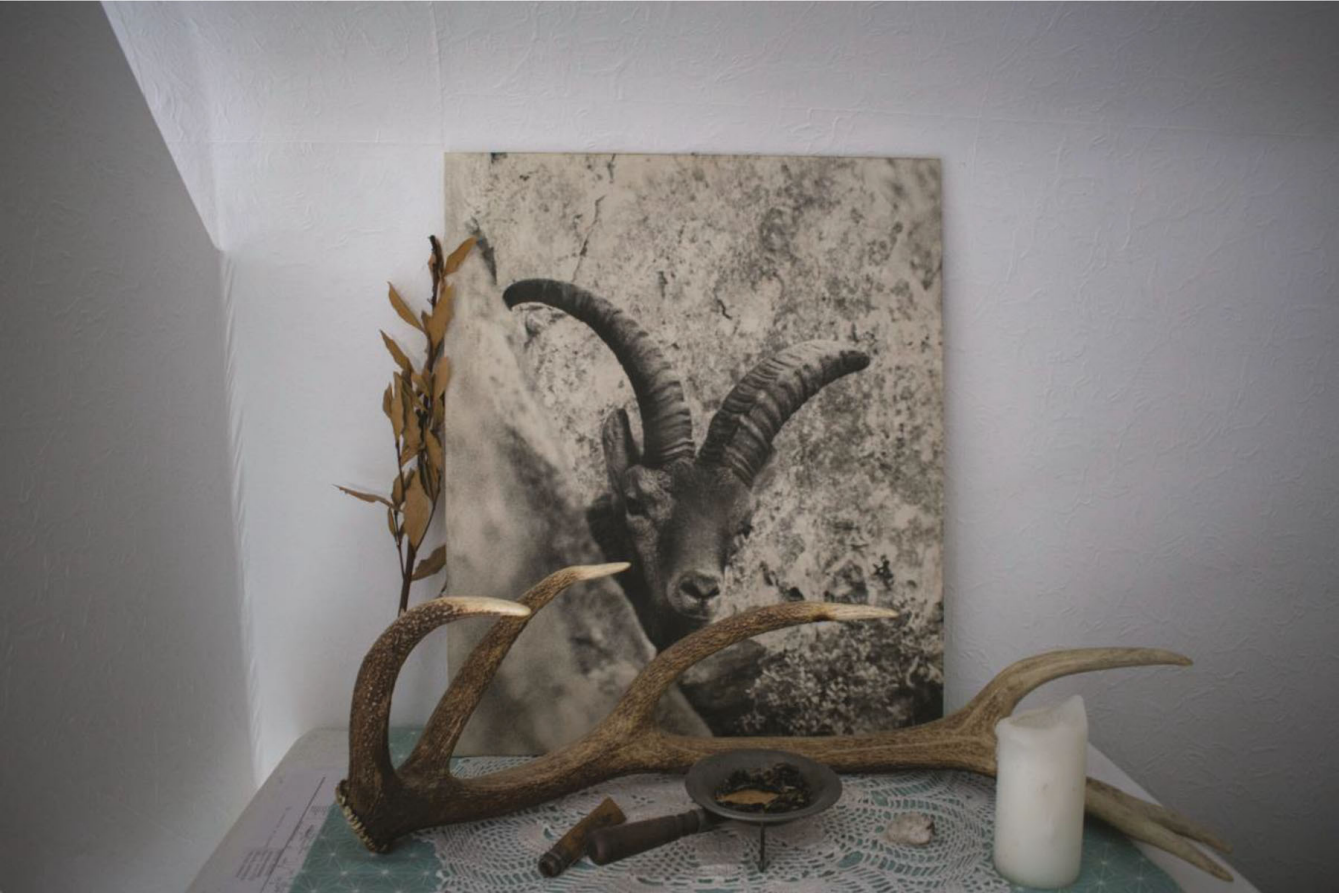
Photographed bucardo by Bernard Clos, in Flo’s bedroom in Argelès-Gazost.

I encountered another of Clos’s photographs in an apartment near Argelès-Gazost in the French Pyrenees (Figure 7). For Flo, who lives here, this image narrates her own “family story,” in addition to illustrating her personal relationships with the “sacred side of the mountains.” One of Flo’s relatives bought the photo in the early 1980s. This image actively transported Flo to other spatiotemporalities: “I was less than ten years old, and I clearly remember spending a lot of time looking at the ibex in the picture; my grandfather only told me that they were very rare animals that lived in the Pyrenees but nothing more.” This image reconnects Flo to a childlike awe and enchantment, one where “nature is so exhilarating”; now, “the photo of the ibex for me is the energy of the mountain.” The image, for Flo, “creates a sense of being a little scared; the ibex has an intense look, at the same time half-hidden behind the rock. It feels unattainable but also mystical. It takes me back to my childhood fascination. But also, when I look at this photo, I feel the emotion as when I am on the mountain peaks, something very mystical that I only found in the Pyrenees.” This image invites speculation—Flo never saw a living bucardo but was able to “dream about them.”

For myself, Juan, and Flo, these photographs allowed us to conjure our own bucardo in the Pyrenean landscape. They act as “convocation apparatuses” (Despret 2019), encouraging us to form mediated relations, forging caring relations through time, space, and across borders of life and death. As mediated objects, these images produce affective atmospheres that encourage the remote sensing of extinct animals through space and time (see McCormack 2010; Despret 2019). These affects are felt differentially across bodies, and thus narrate emotive stories of death, absence, nostalgia, loss, and reverence for the personal reflections that surround the photographs—lending themselves to the liminality of absence and presence. Photographs evidence a presence—a material encounter—and explore the transformative absences left in extinction’s wake. In the bucardo’s case, the recent-ness of these encounters and extent of traces matter. Compared to charismatic extinct fauna like the dodo or mammoth (who, incidentally, are both de-extinction candidate species), these photographs complement embodied experiences with both living animals prior to their extinction, and the landscapes in which these more-than-human relations are culturally and historically situated.

**Figure 8.**
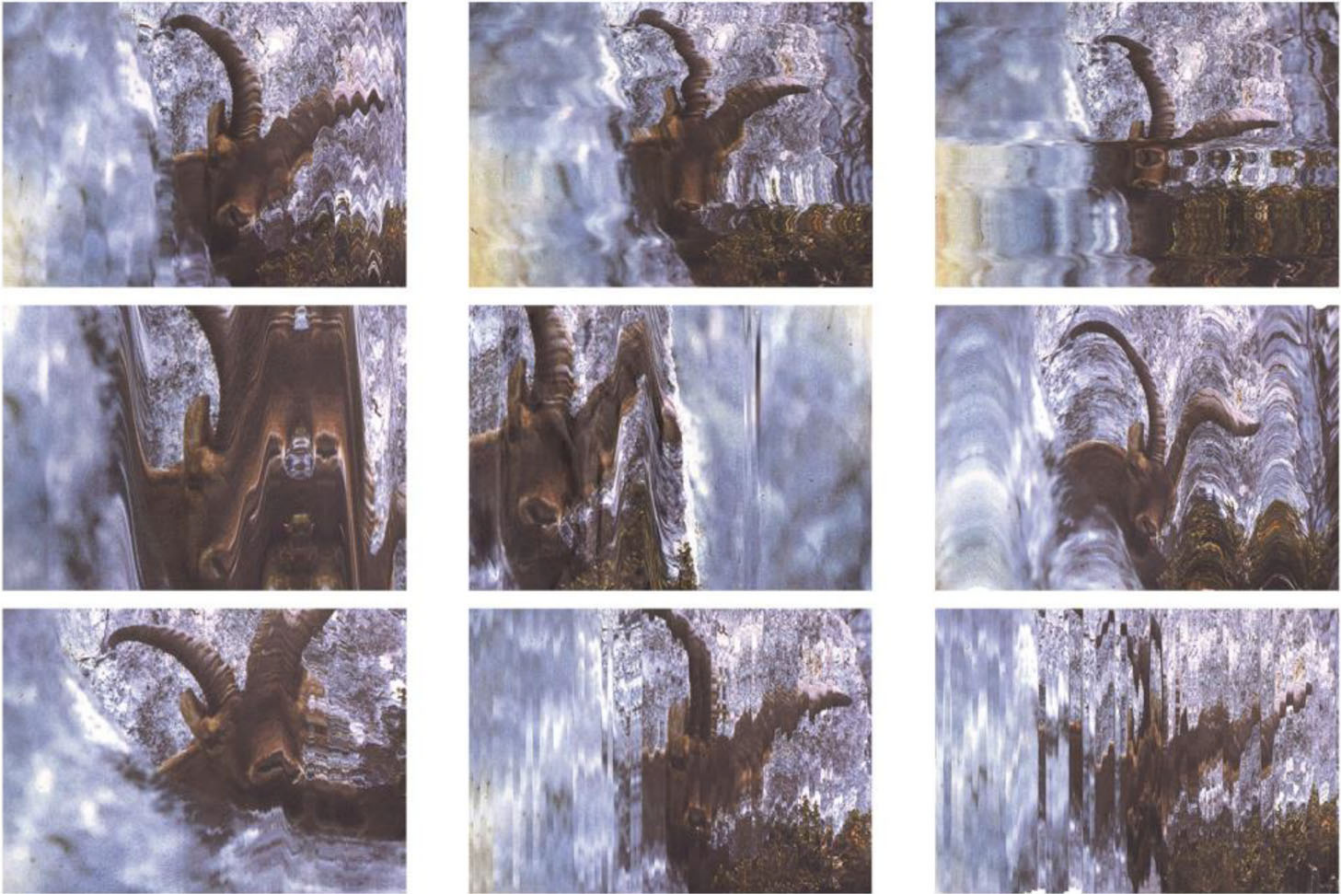
*Bucardo Glitch Series* (2020) by artist Dan J. Towns, mixed media.

In 2020, I revisited Clos’s photography with graphic artist Dan Towns. Dan ran the bucardo photograph through a broken photocopier, playing with the novel forms it produced, and allowing the agency of the technology itself to narrate this visual story. The colors ran and the images fell out of place, glitched, and jumped around. Each new copy becomes further distorted from the original, but with close attention, between the grains and hitches, the bodily forms are still there. There, but not there, different, ghostly. Both uncannily unnerving and familiarly comforting. The outcome, *Bucardo Glitch Series* (Towns 2020), addresses the liminal being of extinct animals as cultural symbols, productive of dynamic relations (Figure 8).

**Figure 9.**
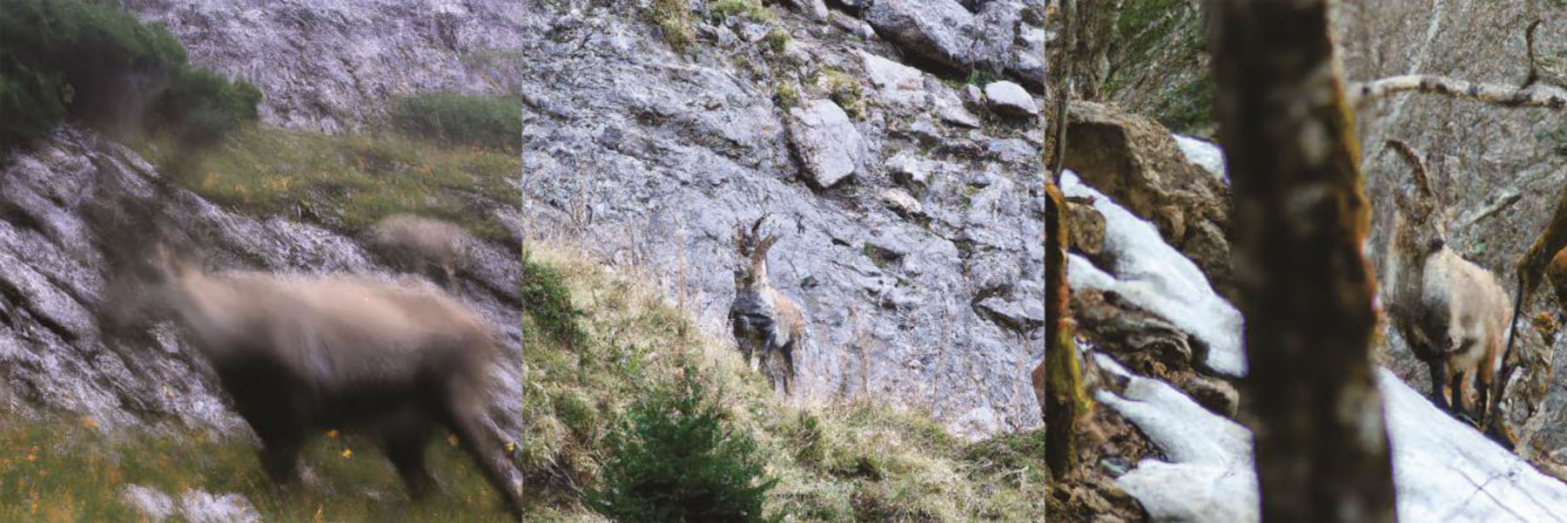
Composite artificial intelligence imagery from *The Last Bucardo* (2022), Christian Kosmas Mayer with Adam Searle, mixed media.

Speculative imaging technologies aided by machine learning programs have proliferated in the early 2020s, exemplifying technological agencies in the reanimation of extinct biota in addition to their collection. In the conceptual multimedia piece, *The Last Bucardo*,^8^ Christian Kosmas Mayer conjures images of hypothetically “deextincted” bucardo in their historical landscape using artificial intelligence. In accessing known bucardo photographs freely accessible online—most of which were taken by Clos in the 1980s—the program hallucinates a series of depictions in which bucardo are reimagined in the Pyrenees. The composite images are ghostlike, uncanny, and unsettling; these bucardo are not the same, but rather technologically mediated fabrications (Figure 9).

Both these artworks pose a series of questions regarding the authenticity of de-extinction projects: To what extent can a cloned animal become a reliable reproduction of absent biota, given the altered cultural milieu in which it emerges? What happens when technology re-creates an ontologically distinct encounter—a semblance of the original but in another political and ecological world? How authentic are reproductions produced by imperfect technologies such as those used in interspecific somatic cell nuclear transfer? The ecological and ethical merits of a revisited bucardo cloning program are disputed given that the biological material of a single individual remains cryopreserved, thus rendering repopulation efforts impossible without hybridization with extant species (García-González and Margalida 2014; Searle 2022). Such questions resemble similar debates concerning the authenticity of ivorybill sound recordings to represent accurately and authentically the extinct (Hunter 2023a), and exemplify the affective, situated, and intersubjective nature of this existential question. Towns and Mayer both prompt considerations of how more-than-human relations are altered and reimagined—across affective and epistemological registers—as they transcend spatial and temporal scales. The metamorphic nature of these interventions plays with mediating technologies while highlighting their boundedness in natural cultural contexts. It speaks to the experimental and experiential potentials of fabulation and the abilities of real relations to emerge with the material-semiotic traces of nonhumans.

## Conclusions: Speculative Geographies after Extinction

Neither these relations nor our methods here are, of course, wholly novel: To access and story past worlds and lives through their traces defines the project of historical geography. Indeed, people have worked to conjure presences with dead animals for generations: They have animated archives for centuries (Fudge 2013). In addition, there are multiple other forms of extinction’s afterlives beyond the audiovisual that extend beyond the scope of this article, such as the increasing prominence of preserved biological material in conservation cryobanks (Wrigley 2023). Moreover, individual animal afterlives unrelated to extinction proliferate in both agriculture and conservation, where extracted biological matter leads a material and political afterlife of its own (e.g., Colombino and Giaccaria 2016). We contend, however, that the entanglement of extinction with audiovisual traces demands distinct geographical consideration and a distinct geographical response. In conclusion, we consider our two cases in conversation, demonstrating how extinction’s audiovisual afterlives, in their storied assemblages, can and might affect more-than-human geographies.

Extinction itself is an unmistakably charismatic phenomenon—a powerful story that propels much environmental discourses and action. It is not only that the ivorybill and bucardo are animated after extinction through audiovisual traces, but these traces themselves are animated in the context of extinction. Recorded media always call “forward in time to an anticipated viewer” (Schneider 2011, 140)—mediated encounters remain agential through circulation and demanding response in place. In our cases, human practices sought to capture elements of animal life prior to individual death—including photography and sound recording—or construct representations of life through reworked biological remains—including taxidermy and cryopreservation. These interventions shared a common concern in creating opportunities for future generations to know, attune to, and speculate on relations with absent biota. Our place, as more-than-human geographers researching and living through ecological catastrophe, demands a specific response to these mediated remains of extinction, bounded by historical, cultural, and technological milieus in which they are situated.

In our cases, technologies—like the camera, microphone, headphones, display screen, photograph, and computer memory—acted as “convocation apparatuses” that summoned extinct animals into presence. Within these mediated assemblages, lines between life and death and presence and absence are obscured. For many humans encountering representations of nonhuman life, convocation apparatuses bring presence to absent nonhumans that are extant and can invoke feelings of intimacy and care toward beings that are never encountered in the flesh. Yet in countless examples, convocation apparatuses have broken these relations between species that have been extinct for decades or centuries. Indeed, “if we *don’t* care for them, the dead die stone dead” (Despret 2021d, 5). Lively, mobile animal afterlives like sound recordings and photographs forge complex ecologies spanning disparate spatial and temporal scales. Distance-reducing technologies like these enhance human capacities to relate to, learn from, and be affected by more-than-human life. Although we recognize relationships with technological afterlives are relations in and of themselves, postextinction geographies point to the excess of these, that encountering an animal through such technologies can provoke feelings of solidarity toward the animals represented and the complex knots of multispecies relations they left behind. Thus, in lieu of encounters with living animals, researchers and publics can continue to build deep, world-changing relationships with extinct species. Such encounters hold political potential as an antidote to overwhelming, abstracted narratives about our ecological breakdown, and alter experiences, and even trajectories, of extinction events. Convocation apparatuses call political obligations to the fore: to both historical, extinct nonhumans and human practices, and futures of more-than-human flourishing.

These traces and the stories they facilitate are assemblages through which “we will inevitably be drawn into new connections and, with them, new accountabilities and obligations” (van Dooren and Rose 2016, 90). Our mediated speculations are forms of what Despret (2021c) called “improbable alliances” through time and space: They are often unintentional ways of “allowing a situation to emerge that is going to make alliances possible, visible, comprehensible, and interesting to describe” (32). Extinct animals themselves will remain unaffected through such stories, but such interventions contribute to worlds of living in both negative and positive ways. As Stanyek and Piekut (2010) noted, “being recorded means being enrolled in futures (and pasts) that one cannot wholly predict nor control” (18). For the ivorybill and the bucardo, technologies elicit lively, complex, and contested more-than-human geographies after extinction; they do not narrate a singular reality but allow for multiple realities to coexist.

These extinction narratives show that affective intensities of more-than-human relations are not limited to the discrete moments of life or a reductionist interpretation of what counts as a meaningful encounter. Animals’ traces can provoke “epiphanies” in a human subject—as illustrated in our empirical stories of more-than-human relations—who “looks at things in a different way” as a result (Lorimer, 2007, 922). Through technological reanimation, extinction’s afterlives allow these relations to change across spatial and temporal scales; novel encounters emerge and fundamentally alter understandings of history, experiences of the present, and hopes for the future. These meetings with extinct biota fit with Haraway’s (2008) account of multispecies “contact zones,” where “flesh-to-flesh” encounters between species make a difference, they “are where the action is, and current interactions change interactions to follow … [they] change the subject—all the subjects—in surprising ways” (219). The resulting relations are “organic-technological hybrids” (Haraway 2008) of mediated ecologies; in our cases, rather than flesh-to-flesh encounters, eye-to-photo or ear-to-voice sensory engagements provide contact zones between extant and extinct.

Diffusion is a fundamental affordance of these technologies that create diverse and contested spaces of ongoing relation. Extinction is thus sensed in both active and passive ways, with differential degrees of affective intensities, shaped by the technological prostheses that facilitate their relation with human bodies (Haraway 2008; McCormack 2010). The bucardo’s image sits in Flo’s apartment in the French Pyrenees, as much as it sits within the pages of *National Geographic* magazines, as much as it sits on the digital pages of this article, all sensed and experienced in distinct ways. We can listen to the ivorybill sound recording while we write these words, just as you can listen as you read them, just as the same recording is played out in a forest in Louisiana. Human engagements with these traces occur privately, but are experienced in diffuse and variegated ways across time and space.

It is through such dispersal that the political possibilities of technological animation could be realized: Intimate world-changing encounters are extended by mediation to both the dead and to the collective. The outcomes of these meetings will differ—some will experience epiphanies, some longing, some pain, and some indifference. Given recent attention to the “societal extinction of species” as a driver of biodiversity loss (Jarić et al. 2022), such encounters are noteworthy regardless. As shown through our empirical illustration, mediative technologies in postextinction geographies do not transport humans to past ecologies, but rather enable them to speculate and tell stories otherwise in the present. Indeed, as in the cases we explored here, such technologies might even inspire or be enrolled in the troubling of extinction events themselves.

In both of our cases, the proliferation of digitization has made pivotal changes to how extinct animals are known, circulated, and encountered. Both of us first met these beings through digital circulations of their fractured representations. Initial meetings in magazines and sound archives spurred years of ghost chasing and speculation. Like bucardo photographs hung on bedroom walls, encounters with animals’ traces told stories that lead to further stories, ones in which we ourselves became entangled. These cascades of encounter define the technologies discussed here, those that reduce distance through time and space and enable nonhuman traces to become meaningfully and perennially present. Both already charismatic species in life, the sociocultural weight of the bucardo and the ivorybill has been extended, even intensified, by the lively affordances of their traces. In the Internet age, extinct species like these are reanimated as charismatic subjects of mass media and culture. The geographies of animals’ afterlives have been fundamentally altered, and with them—as our two case studies make clear—personal and societal experiences of extinction. The animal traces are “the same,” but other elements of this more-than-human assemblage—for example, cultural meaning, technological apparatuses, and ecological situ—are distinct.

It is for these reasons we want to push beyond understandings of extinct animals’ remains as requisites to their mourning—attempts to “sever contact” with the dead (Despret 2021d). Rather, in the contexts of technological afterlives, animals’ remains can afford *after-liveliness*: diverse, dispersed, and relational presences that make a difference in the world, that tumble out into places beyond comprehension, that coproduce worlds of their own. To acknowledge the animation of animal traces is to acknowledge that they, like living animals, have agencies exceeding our own desires and structures, that they might disrupt or resist, and that they might demand something of us (van Dooren and Rose 2016). They ask ethical questions of those who encounter them (Bezan and McKay 2022). The bucardo and ivory-billed woodpecker show that animal remains—technologically mediated or not—demand speculative responses from humans: imagining speculative presents of contended absence, or speculative futures of cyborg presence, or speculative relations through geographic research and writing practice.

As we have shown, technologies of reanimation are not neutral. It matters which technologically mediated stories tell stories for relating to the extinct (to paraphrase Haraway 2016). Technologies of capture, collection, categorization, and dissemination are almost ubiquitously entangled with violent systems of extractive and colonial capitalism. For instance, digital archives can be harmful ecologically and socially: Despite their superficially immaterial appearance, they rely on networks of resource-intensive data stores, and the material technologies of collection such as cameras, microphones, and tracking devices are made possible by the extraction of raw materials and often exploitative human labor (see Turnbull et al. 2023). The same channels that facilitate speculative engagements with extinct biota, then, might also exacerbate the struggles of living humans and animals, and even cause further extinctions. Furthermore, extinct animal traces continue to be haunted by the conditions of their collection, which often follow and perpetuate the violence of colonial natural history collection. For instance, early wildlife sound recording expeditions had unmistakable parallels—and even overlaps—with the racist tradition of salvage ethnography and other imperial collecting exploits (Bronfman 2017; Hui 2022).^9^ There is a not-so-subtle irony to scientific endeavors to capture the last representatives of a species (Barrow 2019), subjecting them to new modes of biopolitical governance and apprehending animals “at the historical moment when they are receding from everyday view” on epistemological and ethical grounds (Traisnel 2020, 3).

For all these reasons, it is crucial not to blindly celebrate the possibilities of technological reanimation. We must meet these technologies critically and carefully, refusing to sever their political and world-building potentials from the ecological harms and world destruction they necessitate. We believe the problems of these technologies do not necessarily negate their promises, but rather that realizing the power of mediated relationships demands that these tensions are taken seriously. Such actions might include discouraging the creation of new technologies and long-distance air travel for collecting expeditions, demanding transparency in the data storage processes of digital multimedia archives, and challenging the colonial infatuation with (audiovisual) extraction, categorization, and display (Kohler 2006; Robinson 2020).

Nevertheless, convocation apparatuses facilitate animate postextinction geographies and pose a set of provocations to geographers engaging the mediative qualities of technologies in more-than-human relations. They highlight the productive and creative potentials of tracing and speculating with assembled afterlives, which contribute to world-changing moments, epiphanies, and multiplicities of knowledge. To paraphrase McCormack’s (2010) haunting repetition: Where is the bucardo? Where is the ivorybill? Our answer: scattered across diverse technocultural assemblages, creating, and exerting power in worlds through their affective and political influence on the living.

## Notes

1 *Ivory-billed woodpecker* or *ivorybill* refers to both Cuban and U.S subspecies (Lammertink and Estrada 1995). Here, we limit our investigation to the U.S. ivory-billed woodpecker.

2 Despret proffered the French *agencement* over Brian Massumi’s translation to *assemblage* for etymological links to agency. Here we employ assemblage due to its prominence in Anglophone geography.

3 As McCormack (2010) noted, there are important distinctions to be made between affect, feeling, and emotion. Extinction’s affective afterlives could be obscure but sensed at any moment; these affects are differentially felt across human and nonhuman bodies; and affective intensities can be expressed through emotional frames like loss or nostalgia.

4 Despite the biological phenomenon of “dark extinction” in which species are driven to extinction before they are named and taxonomically categorized (Boehm and Cronk 2021), there exists a richness of oral histories and other accounts of nonhuman life and death outside of Western culture and knowledge politics (see Ibbotson 2017; Wehi et al. 2018).

5 You can listen here: https://macaulaylibrary.org/asset/6784#_ga=2.49494630.1919948211.1656352712-2013034834.1655304350.

6 The Library of Natural Sounds (LNS) was the archive’s original name.

7 The image remains the cover photograph for the online article at https://www.nationalgeographic.com/magazine/article/species-revival-bringing-back-extinct-animals.

8 Adam Searle collaborated with Mayer for this work, which brought together material traces of the bucardo’s afterlives such as the trap that was used to trap it, its taxidermic remains, videos of its cloning, and other forms of visual narration. This was shown at the Deutsches Hygiene-Museum Dresden from February to September 2023, as part of the exhibition *Von Genen und Menschen* (Of Genes and Human Beings).

9 This is not only a historical issue. In the U.S. and Canadian context, for example, many settler scientists, conservationists, and hobbyists continue to collect and capitalize on animal traces from the traditional territories of Indigenous peoples without consideration or permission, land that is often unceded and continues to be disputed (Kanngeiser 2023).
